# Predictive ability of an early diagnostic guess in patients presenting with chest pain; a longitudinal descriptive study

**DOI:** 10.1186/1471-2296-11-14

**Published:** 2010-02-21

**Authors:** François Verdon, Michel Junod, Lilli Herzig, Paul Vaucher, Bernard Burnand, Thomas Bischoff, Alain Pécoud, Bernard Favrat

**Affiliations:** 1 Institute of General Medicine, University of Lausanne, Bugnon 44, 1011 Lausanne, Switzerland; 2 Clinical Epidemiology Centre, Institute of Social and Preventive Medicine, Hospices-CHUV and Faculty of Biology and Medicine, University of Lausanne, Bugnon 21, 1011 Lausanne, Switzerland; 3 Department of Ambulatory Care and Community Medicine, Bugnon 44, 1011 Lausanne, Switzerland

## Abstract

**Background:**

The intuitive early diagnostic guess could play an important role in reaching a final diagnosis. However, no study to date has attempted to quantify the importance of general practitioners' (GPs) ability to correctly appraise the origin of chest pain within the first minutes of an encounter.

**Methods:**

The validation study was nested in a multicentre cohort study with a one year follow-up and included 626 successive patients who presented with chest pain and were attended by 58 GPs in Western Switzerland. The early diagnostic guess was assessed prior to a patient's history being taken by a GP and was then compared to a diagnosis of chest pain observed over the next year.

**Results:**

Using summary measures clustered at the GP's level, the early diagnostic guess was confirmed by further investigation in 51.0% (CI 95%; 49.4% to 52.5%) of patients presenting with chest pain. The early diagnostic guess was more accurate in patients with a life threatening illness (65.4%; CI 95% 64.5% to 66.3%) and in patients who did not feel anxious (62.9%; CI 95% 62.5% to 63.3%). The predictive abilities of an early diagnostic guess were consistent among GPs.

**Conclusions:**

The GPs early diagnostic guess was correct in one out of two patients presenting with chest pain. The probability of a correct guess was higher in patients with a life-threatening illness and in patients not feeling anxious about their pain.

## Background

The decision making process for a General Practitioner (GP) is intuitive and does not merely rely on specific signs. The early intuitive diagnostic guess arises during the initial few minutes of a patient encounter and could also play a role in treatment decisions [1,2], although this simple cognitive strategy may be erroneous [3]. Non-explicit pathways have been shown to be relevant in children with serious infections [4]. General practitioners have also reported using "gut feelings" for referral of patients presenting with chest pain [5]. The presentation of chest pain in a primary care setting has a large spectrum of aetiologies including potentially life-threatening conditions [6-8]. GPs have acknowledged using non-specific signs, such as patients' appearance, in the decision making process for patients presenting with chest pain [5]. However, the importance of this phenomenon has never been quantified. The aim of this study is therefore to determine the importance of an early diagnostic guess by analyzing the association between a correctly suspected early diagnosis and a confirmed final diagnosis in patients presenting with chest pain.

## Methods

### Design

This validation study was nested in a large multicentre observational study exploring the management of thoracic pain in primary care [9,10] in which a random sample of patients seeing a GP for chest pain was included. The early diagnostic guess, which occurred prior to the patients' history being taken, was then compared to the final diagnosis obtained after one year of follow-up.

### Objectives

In patients with chest pain, we measured the prevalence of cases for which physicians had an early diagnostic guess, which was confirmed by further investigations and a one year follow-up. Furthermore, we examined if this predictive ability was influenced by previous encounters with the patient, previous manifestation of similar chest pain, the severity of the illness and by the patients' anxiety, age, and gender.

### General practitioners

Fifty-eight general practitioners (GP)working in private practice and six working as residents in an academic primary care outpatient department in Western Switzerland volunteered to participate to this study (Table 1). For practical reasons, the six supervised residents were grouped under one common code.

**Table 1:** General practitioners' characteristics

Characteristics	Network of GPs N = 57*
**Age**	
30-39 yrs	11 (19.3%)
40-49 yrs	22 (38.6%)
50-59 yrs	24 (42.1%)
**Gender**	
Male	49 (86.0%)
Female	8 (14.0%)
**Years of experience**	
5-10 yrs	24 (42.1%)
11-20 yrs	26 (45.6%)
>20 yrs	7 (12.3%)
**Number of patients included**	
< 10	25 (43.9%)
10-19	25 (43.9%)
≥ 20	7 (12.2%)
**Location**	
Urban	36 (63%)
Rural	21 (37%)

* Supervised residents (n = 6) from the academic outpatient department are not included in this description. Residents attended to 26 patients. Five of them were women; all were below 40 years of age and had less than 10 yrs of experience.

### Patients

Patients sixteen years of age and over reporting any type of chest pain during the first minutes of their visit were consecutively enrolled. The presence of chest pain was ascertained according to the usual practice of each GP. Chest pain due to obvious causes such as trauma or known body metastases was also included and was not necessarily the chief complaint on presentation.

### Early diagnostic guess

Physicians gave their early diagnostic guess after the first minutes of the encounter with the patient. They were asked to complete the initial part of the case report form (CRF) before investigating the patient's history or performing any medical examination. Physicians based their early diagnostic guess on their previous knowledge of the patient, initial contact, and spontaneous presentation of complaint. GPs were free to report the early diagnostic guess in any terms and made no guess if the most probable cause of pain was unclear. Reported diagnoses were then divided into six categories (Table 2).

**Table 2:** Categorisation of reported early diagnostic guess for chest pain.

Categories of chest pain	Unspecific diagnosis	Specific diagnosis	
		Not life threatening	Potentially life threatening
**Musculoskeletal chest pain**	Musculoskeletal chest pain, referred pain, trauma	Chest wall syndrome, Rib fracture, referred shoulder or spine pain	Costal metastasis
**Cardiovascular origin**	Cardiomyopathy, ischemic heart disease	Arrhythmia, acute hypertension, aortic stenosis, mitral stenosis	Stable or unstable angina, myocardial infarcts, acute angina, pulmonary embolism
**Respiratory origin**	Infectious disease, non-infectious disease	Bronchitis, asthma, COPD	Pneumonia, pleurisy, acute asthma
**Digestive origin**	Peptic affection, cancer	Oesophagitis, gastritis, gastric ulcer, oesophageal spasm	Oesophageal cancer, pancreatic cancer, acute cholecystitis
**Psychogenic chest pain**	Anxiety, somatisation	Acute anxiety, panic attack, anxio-depression, somatoform disorder	
**Miscellaneous**		Mastitis, mastalgia, sarcoidosis, herpes zoster, skin infection, chest wall keloid, acute pyelonephritis	

COPD = Chronic obstructive pulmonary disease

GPs also recorded if a diagnosis for a similar complaint was already known and whether the patient was feeling anxious about the pain. The diagnostic guess was recorded at four time points during the initial patient encounter: prior to history taking and physical exam, post history taking, post physical exam, and at the end of the encounter.

### Reference diagnosis

We used the diagnosis eventually retained after one year of follow-up as the definite diagnosis to be contrasted with the early diagnostic guess. An independent panel of physicians confirmed the follow-up diagnosis. Any new investigation, additional tests, reports from hospitals, or specialist referral that occurred after the initial diagnosis made at the end of the initial encounter was considered to reach the definite diagnosis. Adjudicators remained blinded to the early diagnostic guess. For the patients that were lost at follow-up, the information collected during the study and the patient's up to date medical records were used by the adjudicators. This method is not believed to be perfect but is the best acceptable solution for studies in family practice settings [11]. Quality control of the reported diagnosis was done using patients up to date medical records at the GP's office for ten percent of the included patients. All reported diagnoses at the one year follow-up were then categorized by grouping of disorders in the same manner as the initial guess (Table 2). The definition of a severe, potentially life-threatening illness included myocardial infarction, stable or unstable angina, pulmonary embolism, pneumonia and pleurisy, acute asthma, and neoplasm.

### Statistical methods

Prevalence of cases for which the early diagnostic guess was to be confirmed was calculated at a cluster level for each GP. Summary measure for all GPs was given using a frequency weighed mean value with a 95% CI. Influence of age (<50 yrs vs. >50 yrs), sex (male vs. female), known vs. unknown patient, new vs. known manifestation of chest pain, severe vs. non severe illness and patient anxiety over the predictive ability of early diagnostic guess were estimated. Predictive ability was calculated stratifying the results for each of these variables. Odds of correctly diagnosing the illness early were calculated using random effect logistic regression, adjusting for cluster effects verified by quadrature check. Homogeneity of these effects across GPs was verified by calculating the intraclass correlation coefficient (ρ). No correction for multiple testing was planned; significance level was set at p < 0.05. The study protocol was approved by the official Ethical Commission of Internal Medicine (Prot. 41/2000).

## Results

Among 24,620 consecutive primary care encounters within 59 different general practices, 672 patients presenting with thoracic pain (main or ancillary symptom) were included in the study during five weeks in 2001. Follow-up was 100% at 3 months and 96% at one year. Predictive ability of early diagnostic guess was similar between patients with full data and those with missing data. Patients with missing data were excluded, and one GP with incomplete CRFs was not included in the analysis, leaving 626 (93.2%) patients and 58 GPs. During quality control, only one final diagnosis was contested and resolved after discussion between the GP and the adjudicators. A mean of 12 (SD = 7) patients with chest pain were seen by each GP who participated to the analysis. Their median number of years of practice was 14 yrs (ranging from 1 to 24 yrs) (Table 1).

**Figure 1:**
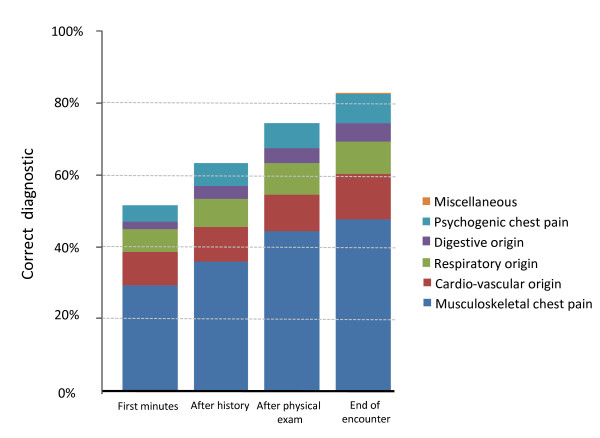
**Prevalence of suspected chest pain origin at different times during the encounter**.

A very slight majority of women were seen with chest pain (51.4%); 51.9% of patients were over 50 yrs. Physicians suspected an illness and reported a specific early diagnostic guess for 441 patients (70.4%); they did not make a guess for 185 patients. GPs' early diagnostic guess was confirmed for 319 patients (51.0%; CI 95% 49.4% to 52.5%) presenting with chest pain. After history taking, this prediction had an absolute improvement of 11.7%, after physical examination of 22.9%, and at the end of the first encounter of 30.9% (Figure 1). A GP's early prediction ability was greater for patients with life threatening illness (65.4%; CI 95% 64.5% to 66.3%) and for those without anxiety (62.9%; CI 95% 62.5% to 63.3%). Early diagnostic guess was predictive for previously unknown patients (47.4%; CI 95% 46.1% to 48.7%) and for patients with a new complaint (48.5%; CI 95% 48.1% to 48.9%). The magnitude of the associations between these factors and the ability to correctly guess the diagnosis (Table 3) seemed homogenous across GPs (ICC; ρ < 0.1), however we observed a non-significant trend for more experienced physicians (≥ 10 yrs practice) showing a greater ability to correctly predict the final diagnosis (OR = 1.5; CI 95% 0.89 to 2.5).

**Table 3:** Physicians' ability (c = 58) to correctly guess the diagnosis for different patients' factors (n = 626).

	Mean physicians' predictive ability	Odds Ratio adjusted for cluster effects		Differences between GP
	% (SD)	OR_CE _(95% CI)	p-value	ρ*
Patients' age				
<50 yrs (n = 257)	56.4% (1.8%)	1.4 (0.98 to 2.0)	p = 0.066	ρ = 0.096
≥50 yrs (n = 369)	50.4% (1.3%)	-		-
Patients' gender				
Men (n = 304)	50.7% (1.5%)	0.93 (0.67 to 1.3)	p = 0.680	ρ = 0.089
Women (n = 322)	55.0% (1.5%)	-		-
Known patient				
Yes (n = 569)	53.1% (0.9%)	1.3 (0.72 to 2.4)	p = 0.386	ρ = 0.088
No (n = 57)	47.4% (4.9%)	-		-
New Complaint				
Yes (n = 301)	48.5% (1.5%)	0.78 (0.56 to 1.1)	p = 0.158	ρ = 0.088
No (n = 325)	54.2% (1.2%)	-		-
Patient feeling anxious				
Yes (n = 348)	44.8% (1.3%)	0.46 (0.33 to 0.65)	p < 0.0001	ρ = 0.090
No (n = 278)	62.9% (1.5%)	-		-
Life threatening				
Yes (n = 104)	65.4% (3.5%)	1.8 (1.1 to 2.8)	p = 0.015	ρ = 0.087
No (n = 522)	50.3% (0.1%)	-		-

* ρ is the intraclass correlation coefficient (ICC) which corresponds to the proportion of the total observed variance which is related to the lack of independence between measures taken from the same physician. A value of 0 indicates no differences between physicians whereas a value of 1 indicates that the associations are totally dependent on physicians.

## Discussion

### Overview of results

In primary care patients presenting with chest pain, we found that half (51%) of the early diagnostic guesses made after the first minutes of the encounter were concordant with the definite diagnosis retained after 12 months. Apparently, the patient's complaint and non-verbal communication play an important role in the decision making process. Predictive ability of the early diagnostic guess was higher in patients with severe illness (65.4%) and for those who did not express anxiety (62.9%). It is important to note that even if the majority of diagnoses are correctly predicted by what has been referred to as "gut feelings" [5], other elements of medical investigations play a crucial role. They contribute to an increase in appropriate diagnosis of over 80%, which is consistent with other observations [7].

### Strength and weakness

To our knowledge, this is the first quantitative study to examine the performance of the first diagnostic impression for patients presenting with chest pain. Our observations should be consistent with what general practitioners experience in their daily work as study data was collected from multiple private practices with a variety of physicians and a large patient sample size. Following patients over one year made it possible to improve the validity of the final diagnosis compared to the diagnosis reached at the end of the first encounter.

A study design limitation is the absence of randomization to select a representative sample of GPs. However, we observed that the 58 general practitioners from our study showed similar traits to those sampled in Western Switzerland in 2004 [12]. Also, the validity of the final diagnosis is not certain. For chest pain, causes can be multiple. Distinguishing chest wall syndromes from psychogenic pains may be difficult as this type of disorder often results from complex psycho-bio-social conditions in which a single cause cannot be clearly identified [13]. Finally, we cannot exclude the possibility that physicians behaved differently during the initial patient encounter due to the additional paperwork and distraction (Hawthorne effect).

### Previous studies

Early diagnostic guess may be obtained by means of non-explicit pathways, based on intuition, associations with stored information and pattern recognition [5]. The first minutes of an encounter include a large amount of information that can be observed without questioning the patient. Patients who show an unusual way of walking, breathing, or moving; those who are sweating, those who are pale, those who appear anxious, or those for whom relatives seem anxious could also influence the physician's decision [4]. Apparently, physicians synthesize informal factors into a global impression that could help them identify that something is not right and improve their ability to correctly identify serious diseases. Qualitative studies have also shown that physicians rely on many non-specific signs in their decision making process [5,14]. The decision making process is more than just a combination of signs and symptoms. It includes a "gut feeling"; a process difficult to dissect [15].

## Conclusion

The GPs' early diagnostic guess was correct in one out of two patients presenting with chest pain. The probability of a correct guess was higher for life threatening illness and in patients without anxiety concerning their pain.

## Abbreviations

CI 95%: confidence Interval of 95%; COPD: chronic obstructive pulmonary disease; CRF: case report form; GP: general practitioner; ICC: intraclass correlation coefficient.

## Competing interests

BF has taken part in advisory board meetings and received honoraria to speak at meetings of drug companies producing drugs to treat iron deficiency. The other authors have no competing interest. We are indebted to the Swiss Academy of Medical Sciences for a grant (RMMA 6/2000).

## Authors' contributions

FV, BF, LH, MJ, BB, TB, AP participated in the conception of the study. FV, BF, MJ, LH and PV planned the statistical analysis. FV, BF, LH, PV, and MJ interpreted the results. FV, BF, LH, and PV drafted and revised the manuscript. All authors have read and approved the manuscript. They had full access to all of the data (including statistical reports and tables) in the study and can take responsibility for the integrity of the data and the accuracy of the data analysis.

## Pre-publication history

The pre-publication history for this paper can be accessed here:

http://www.biomedcentral.com/1471-2296/11/14/prepub

## References

[B1] Greenhalgh T Intuition and evidence--uneasy bedfellows? Br J Gen Pract 2002 52 478 395 400 12014539 PMC1314297

[B2] Tracy CS Dantas GC Upshur RE Evidence-based medicine in primary care: qualitative study of family physicians BMC Fam Pract 2003 4 6 10.1186/1471-2296-4-6 12740025 PMC165430

[B3] Elstein AS Schwartz A Clinical problem solving and diagnostic decision making: selective review of the cognitive literature BMJ 2002 324 7339 729 732 10.1136/bmj.324.7339.729 11909793 PMC1122649

[B4] Bruel A Van den Aertgeerts B Bruyninckx R Aerts M Buntinx F Signs and symptoms for diagnosis of serious infections in children: a prospective study in primary care Br J Gen Pract 2007 57 540 538 546 17727746 PMC2099636

[B5] Bruyninckx R Bruel A Van den Hannes K Buntinx F Aertgeerts B GPs' reasons for referral of patients with chest pain: a qualitative study BMC Fam Pract 2009 10 55 10.1186/1471-2296-10-55 19646225 PMC2731044

[B6] An exploratory report of chest pain in primary care. A report from ASPN J Am Board Fam Pract 1990 3 3 143 150 2378253

[B7] Buntinx F Truyen J Embrechts P Moreel G Peeters R Chest pain: an evaluation of the initial diagnosis made by 25 Flemish general practitioners Fam Pract 1991 8 2 121 124 10.1093/fampra/8.2.121 1874355

[B8] Svavarsdottir AE Jonasson MR Gudmundsson GH Fjeldsted K Chest pain in family practice. Diagnosis and long-term outcome in a community setting Can Fam Physician 1996 42 1122 1128 8704488 PMC2146490

[B9] Gencer B Vaucher P Herzig L Verdon F Ruffieux C Boesner S Burnand B Bischoff T Donner-Banzhoff N Favrat B Ruling out coronary heart disease in primary care patients with chest pain: a clinical prediction score BMC Med 8 1 9 10.1186/1741-7015-8-9 20092615 PMC2832616

[B10] Verdon F Herzig L Burnand B Bischoff T Pecoud A Junod M Muhlemann N Favrat B Chest pain in daily practice: occurrence causes and management Swiss Med Wkly 2008 138 23-24 340 347 18561039 10.4414/smw.2008.12123

[B11] Knottnerus J Buntinx F The Evidence Base of Clinical Diagnosis: Theory and Methods of Diagnostic Research BMJ Books 2009 2 London: Blackwell Publishing

[B12] Monnier M Médecins de premier recours en Suisse romande: Qui sont-ils? Que font-ils? Primary Care 2004 4 41 782 784

[B13] Krieger N Theories for social epidemiology in the 21st century: an ecosocial perspective Int J Epidemiol 2001 30 4 668 677 10.1093/ije/30.4.668 11511581

[B14] Hani MA Keller H Vandenesch J Sonnichsen AC Griffiths F Donner-Banzhoff N Different from what the textbooks say: how GPs diagnose coronary heart disease Fam Pract 2007 24 6 622 627 10.1093/fampra/cmm053 17971349

[B15] Croskerry P A universal model of diagnostic reasoning Acad Med 2009 84 8 1022 1028 10.1097/ACM.0b013e3181ace703 19638766

